# A New Species of *Microhyla* (Anura: Microhylidae) from Nilphamari, Bangladesh

**DOI:** 10.1371/journal.pone.0119825

**Published:** 2015-03-25

**Authors:** Mohammad Sajid Ali Howlader, Abhilash Nair, Sujith V. Gopalan, Juha Merilä

**Affiliations:** 1 Ecological Genetics Research Unit, Department of Biosciences, University of Helsinki, Helsinki, Finland; 2 Molecular Ecology Laboratory, Rajiv Gandhi Centre for Biotechnology, Thiruvananthapuram, Kerala, India; Trier University, GERMANY

## Abstract

A new species of *Microhyla* frog from the Nilphamari district of Bangladesh is described and compared with its morphologically similar and geographically proximate congeners. Molecular phylogeny derived from mitochondrial DNA sequences revealed that although the new species – designated here as *Microhyla nilphamariensis* sp. nov. – forms a clade with *M*. *ornate*, it is highly divergent from *M*. *ornata* and all of its congeners, with 5.7 – 13.2% sequence divergence at the 16S rRNA gene. The new species can be identified phenotypically on the basis of a set of diagnostic (both qualitative and quantitative) characters as follows: head length is 77% of head width, distance from front of eyes to the nostril is roughly six times greater than nostril–snout length, internarial distance is roughly five times greater than nostril–snout length, interorbital distance is two times greater than internarial distance, and distance from back of mandible to back of the eye is 15% of head length. Furthermore, inner metacarpal tubercle is small and ovoid-shaped, whereas outer metacarpal tubercle is very small and rounded. Toes have rudimentary webbing, digital discs are absent, inner metatarsal tubercle is small and round, outer metatarsal tubercle is ovoid-shaped, minute, and indistinct.

## Introduction

Species delimitation is an important element in both ecology and evolutionary biology research, and in particular, in the development of biodiversity management strategies and plans [1]. However, species diversity in many organismal groups is still poorly documented, even in some vertebrate taxa such as amphibians {e.g. [2, 3]}. This is especially true in areas with a high degree of endemism, such as the Western Ghats biodiversity hotspot of India (e.g. [4, 5]), but also in adjacent areas such as Bangladesh, from where new species are being described at an increasing rate [6, 7]. In general, amphibians from Southern Asia are poorly studied, but recent genetic studies have identified many cryptic species from multiple genera and families {e.g. [5, 8]}. Southern Asia has very high amphibian diversity, which is particularly prominent in families such as Dicroglossidae and Microhylidae. Additionally, many new cryptic species continue to be identified in these families, particularly from Bangladesh [9, 10], hinting at the possibility of many more species within these families to become recognized in these regions [10, 11].

*Microhyla* is one of the Asian genera in the frog family Microhylidae, comprised of 38 known species [12]. In Southern Asia, there are currently nine recognized species belonging to this genus [13], each having very different distribution ranges in this region. For example, *M*. *heymonsi* and *M*. *fissipes* were initially described from Taiwan [12, 14, 15], but *M*. *heymonsi* has also been reported from Northeast India [16], and *M*. *fissipes* from Bangladesh [6]. *M*. *berdmorei* was described from Myanmar [17], and later reported from Northeast India [16] and Bangladesh [18]. *M*. *karunaratnei* and *M*. *zeylanica* are species endemic to Sri Lanka [19, 20]. *M*. *sholigari* is restricted to South and Southwestern India [21, 22] and has been suggested to be closely related to other Southeast Asian *Microhyla* [21]. *M*. *rubra* and *M*. *ornata* were described from the Western Ghats-Sri Lanka biodiversity hotspot [22–24], which represents an area hosting major endemic radiations of amphibians [25–27]. In contrast to other parts of South Asia, Microhylids of Bangladesh have remained largely unstudied. However, recent studies have discovered that several Microhylid frogs from Bangladesh do not belong to the species category to which they were initially assigned on the basis of morphological criteria [6, 11]. For example, *M*. *rubra* and *M*. *ornata* described from South India have also been reported from Bangladesh based on photographic comparisons, though recent evidence from molecular studies refutes their presence in Bangladesh [6]. Bangladeshi populations of *M*. *berdmorei* are also currently documented as unnamed species on the basis of high genetic divergence from samples collected from the type locality [6]. Similarly, *M*. *ornata* is believed to be a member of the complex species group that contains several undescribed species [6, 10, 11]. Hasan *et al*. [10] described two new species, *M*. *mymensinghensis* and *M*. *mukhlesuri*, which bear strong morphological resemblance to the original description of *M*. *ornata* [12], though both of the species are genetically more closely related with *M*. *fissipes* than with *M*. *ornata*. Moreover, both Matsui *et al*. [10] and Hasan *et al*. [6] reported an unidentified species from Dinajpur district of Bangladesh that is highly genetically divergent from *M*. *ornata* collected from the type locality in South India. To confirm their inference, we have sequenced (see results) *M*. *ornata* from the type locality in the Western Ghats, India and compared it with sequences of unidentified specimens of *Microhyla* collected from Dinajpur in Bangladesh. In the present study, we found the same haplotypes of the unidentified *Microhyla* species reported by both Matsui *et al*. [10] and Hasan *et al*. [6] from Nilphamari, which is ~100km away from their initial collection localities. We also compared our specimens with *M*. *ornata* specimens collected from the type locality in the Western Ghats and preserved in the Zoological Survey of India (ZSI). The comparison of the *Microhyla* species from Bangladesh with the specimens from ZSI uncovered significant morphological differences. Herein we present the formal description of the new species of *Microhyla*, with genetic and morphological comparisons to other species in the genus.

## Materials and Methods

### Ethics Statement

This study was conducted with appropriate permissions (CCF letter no. 22.01.0000.101.23.2012.681 for collecting specimens, CF memo no. 22.01.0000.101.23.2012 for transport) and guidelines from the responsible authority, the Forest Department, Ministry of Forest and Environment, the People’s Republic of Bangladesh. The protocol of our collection and research were approved by the committee of the Wildlife Section of the Forest Department, Bangladesh, and strictly complied with the ethical conditions as dictated by it and the law of Wildlife Preservation & Security Acts, 2012 (Chapter 10, section 48). Collected specimens were neither recognized as threatened species, nor are they listed in IUCN Redlist or by CITES. All specimens were collected from a small industrial town of northern Bangladesh (25°48′06.12"N, 88°53′59.21"E), which is not considered as a protected area.

### Taxa and specimens

**Fig 1:**
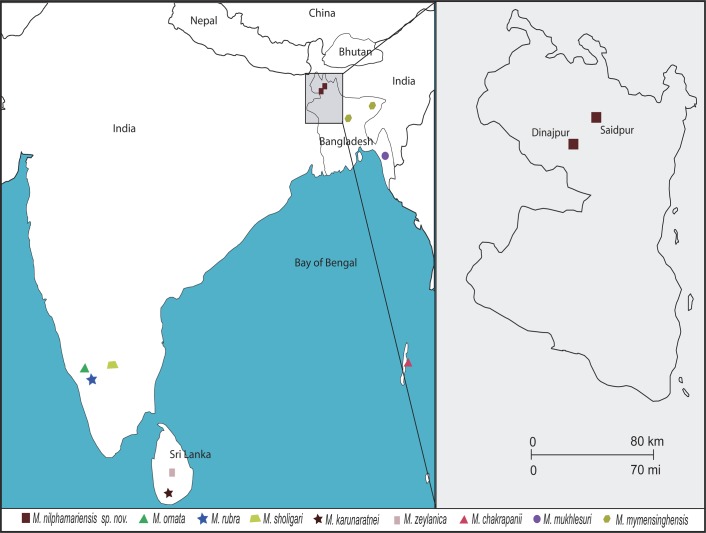
A map showing localities from where *Microhyla nilphamariensis* sp. nov. as well as other *Microhyla* species discussed in this paper have been encountered.

Seven adult specimens (one male and seven females) of *Microhyla sp*. were collected from Koya Golahut, Saidpur, Nilphamari, Bangladesh, in 2012 (Fig. 1). After collection and preliminary identification, live specimens were euthanized. Before fixation, muscle samples were taken from toes and stored in 95% ethanol for subsequent DNA extraction and sequencing. Specimens were fixed in a 10% solution of formaldehyde and subsequently preserved in 75% ethanol for morphological examinations. Holotype and paratopotypes of new species were deposited at the Finnish Museum of Natural History. Additional specimens used for morphological comparisons and their accession numbers are listed in S1 Table. Museum abbreviation include: MZH (Finnish Museum of Natural History), and ZSI (Zoological Survey of India).

### Morphological measurements and analyses

Measurements were taken with digital calipers to the nearest 0.02 mm. Characters were measured following the definitions of Islam *et al*. [7], including the following: SVL (snout-vent length); HL (head length); HW (head width); MN (distance from back of mandible to nostril); SL (snout length); MFE (distance from back of mandible to front of the eye); MBE (distance from back of mandible to back of the eye); IN (internarial distance); IOD (interorbital distance); EN (distance from front of eyes to the nostril); NS (nostril–snout length); EL (eye length); UEW (maximum width of upper eyelid); HAL (hand length); FAL (forearm length); THIGHL (thigh length); TL (tibia length); TFOL (length of tarsus and foot); FOL (foot length); IMTL (inner metatarsal tubercle length). The trait definitions are depicted graphically in S1 Fig. Webbing formula follows that of Glaw and Vences [28].

Morphological comparisons were done using ratios, as well as by using multivariate statistical methods. The ratios were used to allow comparisons to other Southern Asian *Microhyla* species, as well to provide diagnostic criteria for field-identification. The formal multivariate analyses were used to test for morphological differences among the newly described species and its closest morphological and phylogenetic congeners (*M*. *ornata* and *M*. *rubra*). Multivariate analysis of variance (MANOVA) was used to test if species centroids were significantly different, followed by discriminant function analysis (DFA). A principal component analysis (on correlations) and simple bivariate scatterplots (of diagnostic characters) were used to further explore and illustrate morphometric differences among the species. One-way ANOVAs followed by Tukey’s HSD tests were used test if the PC scores differed significantly among species. All statistical analyses were performed using JMP Pro 10.0.2 software (SAS Institute Inc. USA)

### Sequence analysis and phylogeny

Whole genomic DNA was extracted from muscle tissue (N = 7) using a silica-based method [29] and stored at -20°C. PCR amplification and sequencing of the 16S rRNA gene was done with primers F51 (5'-CCCGCCTGTTTACCAAAAACAT-3') and R51 (5'-GGTCTGAACTCAGATCACGTA-3'); Sumida *et al*. [30]. PCR conditions for amplification consisted of 5.72 μl of dH_2_O, 2 μl of 5 × buffer, 0.08 μl of dNTP, 0.2 μl of Phire enzyme (Thermo Fisher), 0.5 μl of each primer and 1 μl of template DNA, in a total reaction volume of 10 μl. The PCR program was comprised of a preliminary denaturation step at 98°C for 30s, followed by 34 cycles of 98°C for 10s, 55°C for 10s, 72°C for 30s, and ended with final extension at 72°C for 1 min. PCR products were purified by using ExoSap IT (USB Corporation, Cleveland, OH, USA) and sequenced at the Institute for Molecular Medicine Finland (FIMM). Sequence ambiguities were edited by aligning forward and reverse reads using the Geneious 5.6.5 program [31]. Final sequences were deposited in GenBank and their accession numbers are provided in S2 Table.

The nucleotide sequences of the 16S gene were aligned with sequences for other *Microhyla* species available from GenBank (N = 21, S2 Table), with ClustalW built into BIOEDIT [32, 33] using the default parameters. The final sequence length used for further phylogenetic analyses was 446 bp. Sequence divergences (uncorrected *p-*values) were calculated using Mega v 5.5.6 [34], excluding the sites with indels. The phylogenetic analyses were performed using Maximum likelihood (ML) and Bayesian inference methods. The GTR + I + G substitution model was selected as the optimal nucleotide substitution model for both methods. For the ML analysis, branch support was evaluated by using 1000 bootstrap replicates [35] as implemented in Mega v 5.5.6 [34]. For the Bayesian analysis, one million generations were run (Markov chain Monte Carlo method) with a sampling frequency of 100, as implemented in MrBayes 3.1.2 [36]. Convergence of the runs was assessed by the average split frequency of standard deviations (<0.01) and by checking the potential scale reduction factors (~ 1.0) for all model parameters. 25% of the trees were discarded as burn-in and the remaining trees were used to generate the 50% majority rule consensus tree and to estimate the Bayesian posterior probabilities.

We estimated the divergence time between the *Microhyla* species by generating a time tree using the program BEAST 1.8.1 [http://beast.bio.ed.ac.uk/]. Our time tree was calibrated by using two nodal constraints that correspond to: (1) *M*. *fissipes* separated from *M*. *mymensinghensis* before 10.53 (5.48–16.95) mya [10] and (2) 1.7 million year old fossil series from the genus *Gastrophryne* (Family: Microhylidae) [37, 38]. In this case, a normal distribution with standard deviation of 0.5 was used to constrain the node leading to *G*. *olivacea* and *G*. *mazatlanensis* as having occurred between 0.72 and 2.68 mya. This calibration point was used as many fossils of *G*. *olivacea* and *G*. *mazatlanensis* have been reported from Pleistocene deposits ranging from 0.24 to 1.8 mya [38]. The divergence time and node ages were estimated using a lognormal relaxed molecular clock in a Bayesian framework. Markov chain Monte Carlo analyses were run for ten million generations, sampled every 1000 generations. We used Tracer 1.5 [http://beast.bio.ed.ac.uk/Tracer] to view the BEAST 1.8.1 output and to verify that all parameters were adequately sampled (effective sample sizes > 200). A burn-in of 1000 was used before summarizing the time trees.

### Nomenclatural Acts

The electronic edition of this article conforms to the requirements of the amended International Code of Zoological Nomenclature (ICZN), and hence the new names contained herein are available under that Code from the electronic edition of this article. This published work and the nomenclatural acts it contains have been registered in ZooBank, the online registration system for the ICZN. The ZooBank Life Science Identifiers (LSIDs) can be resolved and the associated information viewed through any standard web browser by appending the LSID to the prefix "http://zoobank.org/". The LSID for this publication is: urn:lsid:zoobank.org:pub:A1623AB5–002A-4DA4-ADA4–77DA2F199A65. The electronic edition of this work was published in a journal with an ISSN, and has been archived and is available from the following digital repositories: PubMed Central, LOCKSS.

## Results

### Taxonomic treatment

Amphibia Linnaeus, 1758

Anura Fischer von Waldheim, 1813

Microhylidae Günther, 1858

Microhylinae Günther, 1858

*Microhyla* Tschudi, 1838

*Microhyla nilphamariensis* sp. nov. urn:lsid:zoobank.org:act:11E1D35F-7FC1–43C1–8318–747F9FC0C882

#### Etymology

The species name is derived from the name of the type locality Nilphamari, where the type specimens were collected.

#### Holotype

Adult male, MZH-2362, collected from grass-field (25°48′06.12"N, 88°53′59.21"E), Koya Golahut, Saidpur, Nilphamari, Bangladesh; collected by M. S. A. Howlader, June 9, 2012.

#### Paratopotypes

MZH-2360 (adult female), MZH-2361 (adult female), MZH-2363 (adult female), MZH-2364 (adult female), MZH-2365 (adult female), and MZH-2366 (adult female) collected from the same locality as the holotype; collected by M. S. A. Howlader and Abdur Razzaque, June 9, 2012.

#### Diagnosis

*Microhyla nilphamariensis* sp. nov. is characterized by a combination of the following characters: HL 77% of HW, EN roughly six times greater than NS, IN roughly five times greater than NS, IOD two times greater than IN, MBE 15% of HL, small ovoid-shaped inner metacarpal tubercle, very small rounded outer metacarpal tubercle, toes with rudimentary webbing, absence of digital discs, inner metatarsal tubercle small and round, outer metatarsal tubercle ovoid-shaped, minute, and indistinct.

### Description of taxa

#### Holotype (adult male)

Small sized frog (SVL 17.36 mm). Head large, triangular, wider than long, HL 77% of HW, HW 27% of SVL, HL 21% of SVL, MFE 67% of HL, MBE 15% of HL. Snout nearly rounded in lateral view, SL 46% of HL; canthus rostralis indistinct, loreal region concave. Nostrils much closer to snout tip than to eyes, NS 16% of EN; NS 1% of SVL, EN 7% of SVL; nostrils rounded and very small, NS 20% of IN, MN 92% HL. Eye large, EL 52% of HL, EL 11% of SVL; interorbital distance greater than maximum width of upper eyelid greater, UEW 41% of IOD, UEW 44% EL, UEW 4% SVL. Interorbital space convex, IN 49% of IOD. Tympanum is hidden.

Arms moderately long, FAL 74% HAL, FAL 16% of SVL, HAL 22% SVL. Fingers small, free of webbing, tips are flattened. Relative length of fingers, shortest to longest: 1 < 2 < 4 < 3; fingers lacking dermal ridge. Palm with ovoid-shaped inner metacarpal tubercle, small rounded outer metacarpal tubercle. Subarticular tubercle prominent, rounded, single tubercle per digit.

Hind limbs relatively long, TL 42% of SVL, THIGHL 86% of TL; FOL 49% SVL and TL 86% FOL, FOL 71% of TFOL. Toes long, thin, tips rounded; webbing between toes weakly developed [1(1), 2i (1.75), 2e (1), 3i (2.5), 3e (2), 4i (3), 4e (3.25), 5(1.75)]. Relative lengths of toes, shortest to longest: 1 < 2 < 5 < 3 < 4. Inner metatarsal tubercle small and round, present at base of first toe; outer metatarsal tubercle is ovoid-shaped, minute, indistinct; subarticular tubercles well-developed, nearly ovoid-shaped. Dorsal surface smooth with some tiny tubercles on the back and on the sides the body; tiny granules on upper eyelids, loreal, and cloacal region. Dorsal surface of forelimbs, thigh and tarsi glandular. Throat, chest, abdomen and ventral part of thigh and tibia smooth.

**Fig 2:**
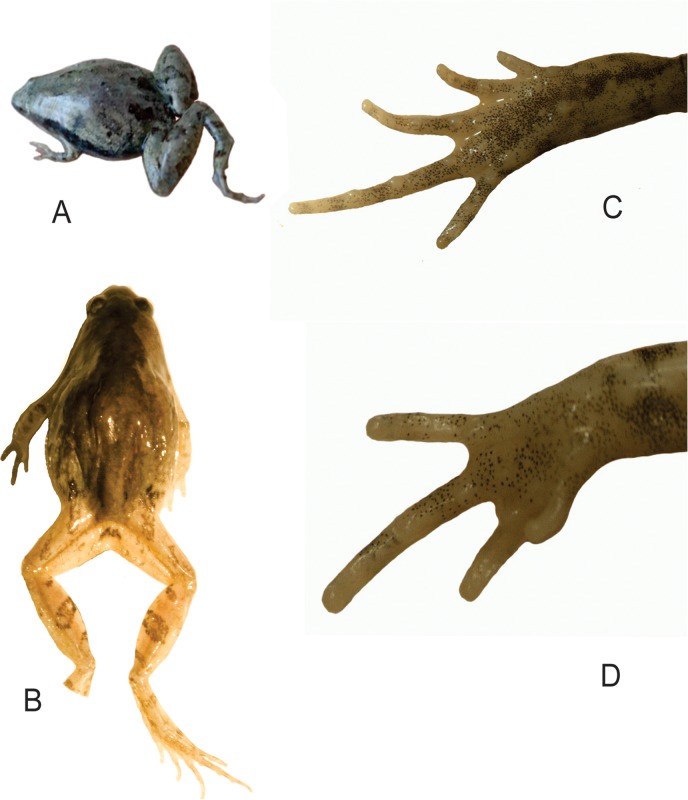
Photographs of *Microhyla nilphamariensis* sp. nov. (A) Dorso-lateral view of male (holotype, live), (B) Dorsal view of male (holotype). (C) Ventral view of foot and (D) palm.

Basic dorsal coloration light brown with distinct dark brown diamond-shaped marking over the back, beginning between the eyes and extending to both the eyelids, narrowing behind the head and widening above the shoulder, then narrowing again and finally broadening out, sending a stripe to the groin and thigh (Fig. 2). A dark streak extends along the sides from back of the eye to shoulder. Limbs with dark cross bars. The belly is dull white; the throat and chest are brown.

#### Measurements (in mm)

Male (holotype): SVL 17.36; HL 3.74; HW 4.82; MN 3.47; SL 1.75; MFE 2.52; MBE 0.57; IN 1.03; IOD 2.09; EN 1.25; NS 0.21; EL 1.95; UEW 0.86; HAL 3.93; FAL 2.91; LAL 1.72; THIGHL 6.41; TL 7.45; TFOL 11.96; FOL 8.61. Female (paratopotype): SVL 17.84; HL 3.85; HW 5.02; MN 3.57; SL 1.79; MFE 2.61; MBE 0.6; IN 1.06; IOD 2.16; EN 1.27; NS 0.22; EL 1.98; UEW 0.89; HAL 4; FAL 2.97; LAL 1.81; THIGHL 6.44; TL 7.51; TFOL 12.07; FOL 8.71.

#### Variation

Morphometric variability is described in Table 1.

**Table 1:** Summary of quantitative and qualitative diagnostic characters in *Microhyla nilphamariensis* sp. nov. and its closest morphological and phylogenetic congeners.

	*Microhyla nilphamariensis* sp. nov.		*Microhyla ornate*		*Microhyla rubra*	
	Male (n = 1)	Female (n = 6)	Male (n = 6)	Female (n = 4)	Male (n = 1)	Female (n = 3)
**HL:HW**	0.77	0.76±0.01	0.95±0.03	0.97±0.02	0.91	0.96±0.01
		(0.74–0.77)	(0.89–0.97)	(0.95–1.00)		(0.95–0.97)
**HL:SVL**	0.21	0.22±0.01	0.28±0.01	0.28±0.02	0.3	0.34±0.01
		(0.21–0.23)	(0.27–0.29)	(0.26–0.30)		(0.33–0.35)
**MBE:HL**	0.15	0.15±0.01	0.45±0.13	0.45±0.15	0.4	0.36±0.01
		(0.14–0.16)	(0.31–0.64)	(0.31–0.59)		(0.35–0.38)
**EL:HL**	0.52	0.52±0.01	0.49±0.03	0.47±0.03	0.52	0.43±0.05
		(0.51–0.54)	(0.45–0.54)	(0.43–0.50)		(0.37–0.46)
**UEW:EL**	0.44	0.42±0.02	0.39±0.03	0.39±0.02	0.4	0.53±0.04
		(0.39–0.45)	(0.34–0.41)	(0.36–0.42)		(0.51–0.57)
**EN:NS**	5.95	5.92±0.08	1.51±0.23	1.60±0.22	1.08	0.97±0.13
		(5.77–5.98)	(1.24–1.92)	(1.43–1.92)		(0.81–1.04)
**IN:NS**	4.9	4.84±0.04	1.68±0.36	1.77±0.41	0.91	1.03±0.12
		(4.91–4.81)	(1.30–1.86)	(1.37–2.32)		(0.89–1.10)
**IOD:IN**	2.03	2.02±0.03	3.09±0.44	2.87±0.23	3.91	3.30±0.09
		(1.98–2.05)	(2.79–3.65)	(2.6–3.12)		(3.19–3.38)
**SL: HL**	0.47	0.47±0.01	0.41±0.02	0.39±0.01	0.4	0.37±0.04
		(0.45–0.49)	(0.38–0.43)	(0.38–0.41)		(0.32–0.39)
**Metacarpal tubercle**	Ovoid-shaped inner metacarpal tubercle; very small rounded outer metacarpal tubercle		Large goblet-shaped inner metacarpal tubercle; very large and prominent, heart shaped outer metatarsal tubercle which appears as two tubercles fusing to form a single one.		Elongated inner metacarpal tubercle; very large and prominent, heart-shaped outer metatarsal tubercle.	
**Metatarsal tubercle**	Inner metatarsal tubercle small round shaped; outer metatarsal tubercle is ovoid, minute, and indistinct.		Inner metatarsal tubercle elongated, large and very prominent; outer metatarsal tubercle is compressed and large in size.		Inner metatarsal tubercle shovel-shaped, bearing longitudinal groove, large and prominent; outer metatarsal tubercle is also shovel-shaped and large.	

Morphological ratios are given as mean ± standard deviation.

#### Distribution

*Microhyla nilphamariensis* sp. nov. is known only from the type locality (Fig. 1). However, Matsui *et al*. [18] and Hasan *et al*. [6] found individuals from Dinajpur district carrying haplotypes similar to those found from the type locality, suggesting that the distribution area might extend beyond the type locality.

#### Natural history

The new species was observed only at night during the rain. At the type locality, specimens were found in a grass-field near temporary pools.

#### Molecular phylogeny and genetic divergence of new species

**Fig 3:**
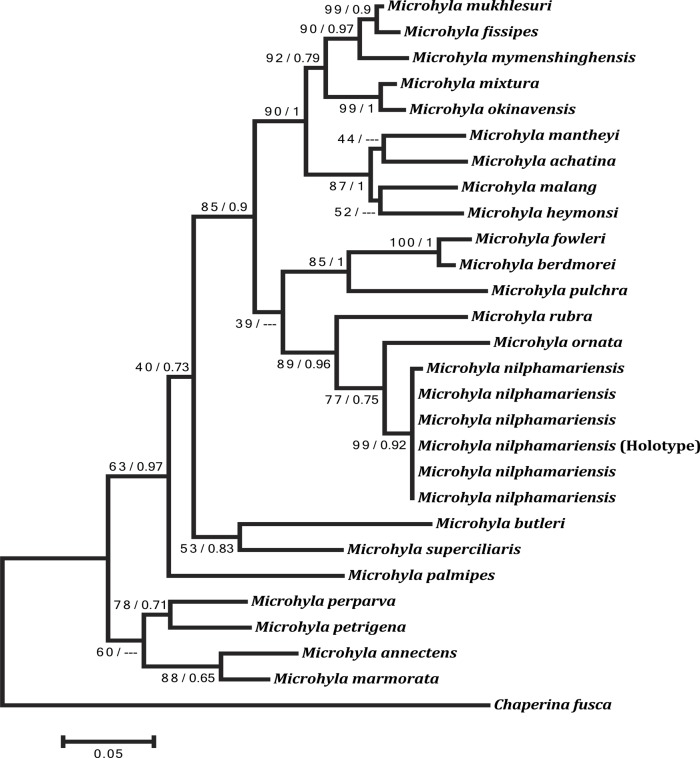
Phylogenetic relationships among all known species in the genus *Microhyla*. Analysis is based on 446 bp of mtDNA (16S gene) sequence, showing the position of *Microhyla nilphamariensis* sp. nov. *Chaperina fusca* was used as an outgroup. The *Microyla ornata* sequence is from Karnataka (Western Ghats, India). Numbers on branches represent bootstrap support values for Maximum-likelihood, and Bayesian posterior probabilities, respectively.

The sequence divergences between *Microhyla nilphamariensis* sp. nov. and other congeneric species were significant, ranging from 5.7% to 13.2% for 16S rRNA (Table 2). Intraspecific genetic divergence within the new species was estimated at 0.5%. *Microhyla nilphamariensis* sp. nov. formed a distinct clade in the phylogenetic analyses with high bootstrap (ML method) and posterior probability support (Bayesian method; Fig. 3). *Microhyla nilphamariensis* sp. nov. was identified as a sister taxa to *M*. *ornata* (Fig. 3). Molecular phylogeny suggests that *M*. *nilphamariensis* sp. nov. belongs to the Indian clade of *Microhyla* species group (including *M*. *ornata* and *M*. *rubra*), rather than having closer affinity to Southeast Asian species (Fig. 3).

**Table 2:** Pairwise genetic divergence (number of base substitutions per site) among *Microhyla* species based on 446 bp mtDNA (16S gene) sequences.

		(a)	(b)	(c)	(d)	(e)	(f)	(g)	(h)	(i)	(j)	(k)	(l)	(m)	(n)	(o)	(p)	(q)	(r)	(s)	(t)	(u)	(v)	(w)
**(a)**	*M*. *nilphamariensis* sp. nov. (Holotype)																							
**(b)**	*M*. *nilphamariensis* sp. nov.	0.005																						
**(c)**	*M*. *ornate*	0.057	0.060																					
**(d)**	*M*. *butleri*	0.123	0.123	0.141																				
**(e)**	*M*. *okinavensis*	0.096	0.096	0.121	0.133																			
**(f)**	*M*. *fissipes*	0.105	0.108	0.110	0.134	0.057																		
**(g)**	*M*. *heymonsi*	0.123	0.119	0.137	0.151	0.090	0.087																	
**(h)**	*M*. *perparva*	0.150	0.150	0.132	0.124	0.140	0.139	0.147																
**(i)**	*M*. *rubra*	0.075	0.082	0.101	0.129	0.128	0.115	0.141	0.152															
**(j)**	*M*. *berdmorei*	0.095	0.102	0.107	0.135	0.107	0.104	0.112	0.139	0.113														
**(k)**	*M*. *mixture*	0.102	0.102	0.120	0.116	0.019	0.057	0.087	0.120	0.122	0.110													
**(l)**	*M*. *malang*	0.110	0.113	0.127	0.133	0.092	0.078	0.065	0.153	0.112	0.116	0.089												
**(m)**	*M*. *achatina*	0.108	0.108	0.120	0.147	0.096	0.090	0.083	0.152	0.124	0.089	0.081	0.077											
**(n)**	*M*. *mantheyi*	0.112	0.112	0.135	0.144	0.088	0.088	0.071	0.146	0.120	0.110	0.079	0.066	0.063										
**(o)**	*M*. *pulchra*	0.114	0.107	0.119	0.134	0.112	0.115	0.121	0.159	0.121	0.086	0.115	0.138	0.115	0.132									
**(p)**	*M*. *superciliaris*	0.114	0.114	0.112	0.099	0.121	0.110	0.129	0.119	0.115	0.125	0.115	0.120	0.133	0.125	0.147								
**(q)**	*M*. *fowleri*	0.095	0.102	0.121	0.138	0.111	0.113	0.116	0.139	0.110	0.021	0.114	0.110	0.089	0.103	0.095	0.129							
**(r)**	*M*. *annectens*	0.132	0.128	0.132	0.130	0.154	0.134	0.145	0.096	0.142	0.100	0.140	0.148	0.142	0.139	0.150	0.123	0.112						
**(s)**	*M*. *palmipes*	0.129	0.129	0.121	0.133	0.144	0.113	0.156	0.110	0.143	0.143	0.129	0.153	0.157	0.155	0.147	0.112	0.142	0.135					
**(t)**	*M*. *marmorata*	0.120	0.120	0.115	0.124	0.128	0.121	0.127	0.081	0.119	0.118	0.115	0.122	0.126	0.127	0.145	0.117	0.121	0.054	0.130				
**(u)**	*M*. *petrigena*	0.130	0.130	0.121	0.137	0.151	0.132	0.132	0.065	0.129	0.120	0.129	0.145	0.137	0.142	0.142	0.115	0.125	0.082	0.110	0.085			
**(v)**	*M*. *mukhlesuri*	0.099	0.103	0.098	0.127	0.055	0.014	0.079	0.130	0.105	0.108	0.055	0.070	0.081	0.089	0.113	0.098	0.118	0.141	0.121	0.115	0.122		
**(w)**	*M*. *mymensinghensis*	0.098	0.098	0.103	0.138	0.059	0.039	0.081	0.129	0.124	0.112	0.053	0.085	0.085	0.089	0.121	0.112	0.116	0.139	0.127	0.114	0.127	0.034	

#### Morphological comparison

**Fig 4:**
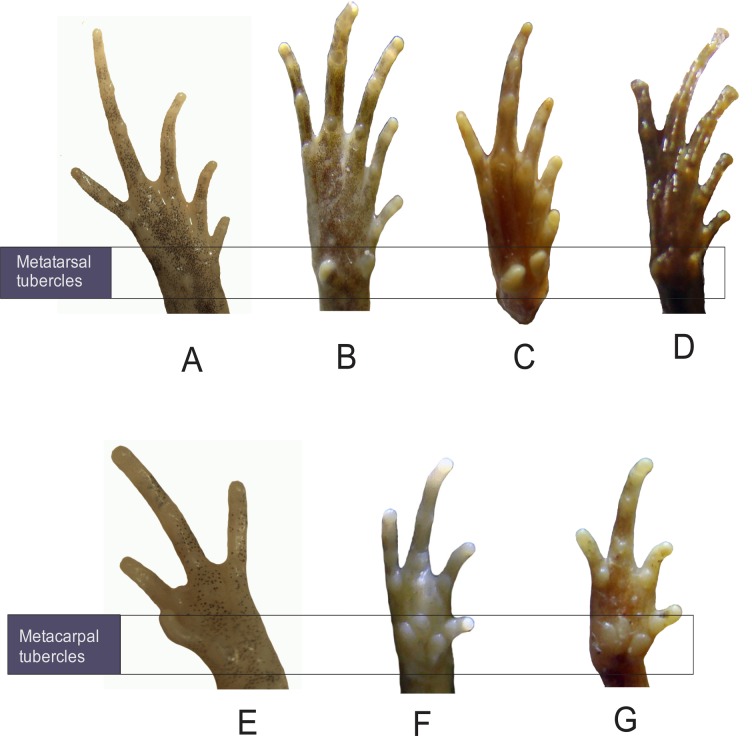
Photographs illustrating diagnostic characters of *Microhyla* species. Ventral views of foot (A) *Microhyla nilphamariensis* sp. nov (holotype), (B) *Microhyla ornata* (accession number: ZSI A9080/2), (C) *Microhyla rubra* (accession number: ZSI A10810/1) and (D) *Microhyla sholigari* (accession number: ZSI A9061). Ventral views of palm of (E) *Microhyla nilphamariensis* sp. nov. (holotype), (F) *Microhyla ornata* (accession number: ZSI A9080/2), and (G) *Microhyla rubra* (accession number: ZSI A10810/1).

**Fig 5:**
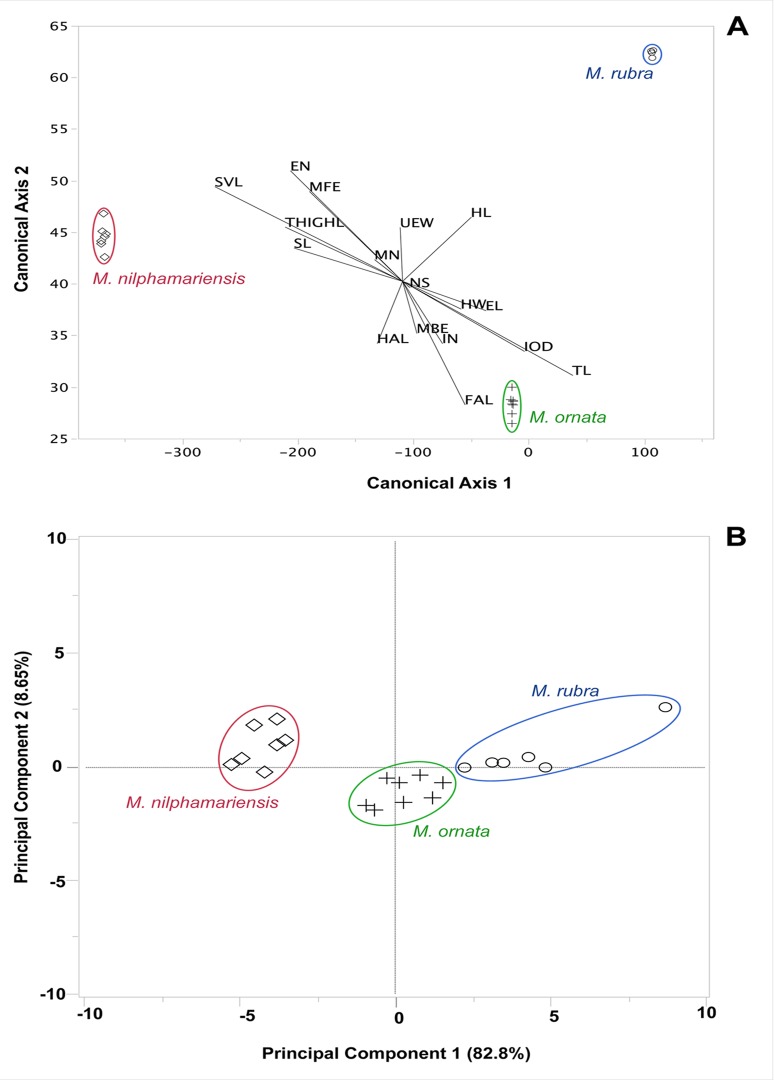
Results of the multivariate analyses of morphometric variability in *Microhyla nilphamariensis* sp. nov., *M. ornata* and *M. rubra*. (A) Discriminant and (B) principal component analysis of morphological traits.

*M*. *nilphamariensis* sp. nov. is morphologically distinct from the closely related species *M*. *ornata* and *M*. *rubra* in the following qualitative characters (Fig. 4): inner metacarpal tubercle small and ovoid-shaped (*vs*. large and goblet-shaped in *M*. *ornata*; elongated in *M*. *rubra*), outer metacarpal tubercle very small and rounded (*vs*. very large, prominent and heart-shaped in *M*. *ornata* and *M*. *rubra*), inner metatarsal tubercle small and round (*vs*. elongated, large and very prominent in *M*. *ornata*; shovel-shaped in *M*. *rubra*), outer metatarsal tubercle ovoid-shaped, minute, and indistinct (*vs*. compressed and large in size in *M*. *ornata*; shovel-shaped and large in *M*. *rubra*). Quantitative diagnostic characters include (see also: Table 1, S2 Fig.): head length 77% of head width (*vs*. roughly equal to head width in *M*. *ornata* and *M*. *rubra*), distance from front of eyes to the nostril roughly six times greater than nostril–snout length (*vs*. over one and a half times greater than nostril–snout length in *M*. *ornata*; over single time greater than nostril–snout length in *M*. *rubra*), internarial distance five times greater than nostril–snout length (*vs*. nearly two times greater than nostril–snout length in *M*. *ornata*; less than two times greater than nostril–snout length in *M*. *rubra*), interorbital distance two times greater than internarial distance (*vs*. more than three times greater than internarial distance in *M*. *ornata* and *M*. *rubra*), distance from back of mandible to back of the eye 15% of head length (*vs*. more than 36% of head length in *M*. *ornata* and *M*. *rubra*). These two closely related *Microhyla* species are morphologically clearly distinct from the new species also according to MANOVA (F_34,18_ = 334.93, P < 0.001), and according to a discriminant analysis which correctly classifies all individuals to their respective species along two significant (Eigenvalues ≥ 194.51, F ≥ 36.47, P < 0.001) canonical axes (Fig. 5A). In a principal component (PC) analysis, the three species are significantly different from each other (Tukey’s HSD; P > 0.05 in all pairwise comparisons) along the first PC-axis (Eigenvalue = 14.07; 82.8% variance explained), which correspond to variation in overall size (all traits loading positively and roughly equally on this axis), *M*. *nilphamariensis* sp. nov. being the smallest species (Fig. 5B). The second PC-axis (Eigenvalue = 1.48; 8.7% variance explained) captures shape differences, but in this axis *M*. *nilphamariensis* sp. nov differs significantly only from *M*. *ornata* (Tukey’s HSD, P < 0.05). Nevertheless, that the *M*. *nilphamariensis* sp. nov. is clearly differentiated from *M*. *ornata* and *M*. *rubra* can also be depicted from bivariate scatterplots (S2 Fig.) showing that it’s diagnostic ratios (see above) do not overlap with those of *M*. *ornata* and *M*. *rubra*.

Genetic divergence of *M*. *nilphamariensis* sp. nov. from all other species in the Southeast Asian clade is 10% to 13% (Fig. 3). Likewise, the new species is morphologically different from all known Southeast Asian species in comparison to the original descriptions [39–55]. Morphological characters of the other Southeast Asian species (*M*. *berdmorei*, *M*. *borneensis*, *M*. *achatina*, *M*. *butleri*, *M*. *palmipes*, *M*. *annamensis*, *M*. *annectens*, *M*. *heymonsi*, *M*. *mantheyi*, *M*. *superciliaris*, *M*. *malang*, *M*. *chakrapanii*, *M*. *marmorata*, *M*. *nanapollexa*, *M*. *pulverata*, *M*. *arboricola*, *M*. *darevskii*, *M*. *minuta*, *M*. *pineticola*, *M*. *pulchella*, *M*. *perparva*, *M*. *mixtura*, *M*. *fowleri*, *M*. *maculifera*, *M*. *petrigena*, *M*. *orientalis*) such as webbed toes with distinct digital discs, and leaf vain-type dorsal surface markings separate them from *M*. *nilphamariensis* sp. nov., which has reduced webbing, absent discs and irregular dorsal surface markings. *M*. *sholigari* also differs from *M*. *nilphamariensis* sp. nov. as it has discs on fingers (Fig. 4). Shovel-shaped inner metatarsal tubercle and a more rounded snout separate *M*. *picta* from *M*. *nilphamariensis* sp. nov. [56]. *M*. *nilphamariensis* sp. nov. lacks the minutely shagreened dorsum, mid-dorsal ridge, and deeply furrowed outer metacarpal tubercle of *M*. *fusca* [57]. *M*. *nilphamariensis* sp. nov. differs from *M*. *pulchra* [58] and *M*. *erythropoda* [59] in having a rounder snout, smaller body size, and rudimentary foot webbing (*M*. *pulchra* and *M*. *erythropoda* have obtuse or obtusely pointed snouts, larger body size and half-webbed toes). *M*. *karunaratnei* [60] and *M*. *zeylanica* [61] are two species endemic to Sri Lanka, and differ from *M*. *nilphamariensis* sp. nov. by extensive digital webbing, presence of digital discs, IOD 1.6 times greater than UEW (*vs*. rudimentary digital webbing, absence of digital discs, IOD 2.43 times greater than UEW). Absence of digital discs differentiates *M*. *nilphamariensis* sp. nov. from *M*. *mukhlesuri*, *M*. *fissipes* and *M*. *mymensinghensis*, in which digital discs are present.

## Discussion

*Microhyla ornata* is considered as one of the most common *Microhyla* species in Bangladesh, exhibiting a high degree of morphological similarity with other species in the genus. The type locality of this species is in the Western Ghats of India (type locality = “Malabar”, Kerala, India) [11, 22, 23], but several new candidate species—formerly recognized as *M*. *ornata*—have been reported from Bangladesh based on genetic information [6, 11]. Both Matsui *et al*. [11] and Hasan *et al*. [6] described a new candidate species from Dinajpur of Northern Bangladesh, based on high mitochondrial DNA (16S rRNA) sequence divergence with the *M*. *ornata* from the Western Ghats (from Karnataka, India). However, no formal descriptions or detailed morphological comparison with other congeneric species were provided for these candidate species. Morphology-based species descriptions are known to be problematic in the genus *Microhyla* because of the high likelihood of homoplasy [62, 63]. Also, the minute body size of *Microhyla* species poses challenges in diagnosing them from their known congeners based on morphology [10, 64]. However, species in this genus are often strongly differentiated in genetic comparisons, facilitating identification of new candidate species. In our study, we have identified a new species that can be clearly differentiated from all know species in the genus *Microhyla*, both by detailed morphological comparisons and by genetic methods. Hence, on the basis of a high degree of genetic divergence and subtle but clear-cut phenotypic divergence from *M*. *ornata*, we have described a new species (*Microhyla nilphamariensis)* and designated a holotype by submitting it to the collections of the Finnish Natural History Museum.

The newly described species grouped genetically with unidentified haplotypes reported as new candidate species by Matsui *et al*. [11] and Hasan *et al*. [6] (S3 Fig.), suggesting that these haplotypes might represent the species we have described in this paper. In fact, GenBank contains several sequences designated as *M*. *ornata*, but for many of them, the collection locality is not specified, and hence, unknown. After aligning all the sequences assigned to *M*. *ornata* from GenBank and conducting a phylogenetic analysis (S3 Fig.), we discovered that many of them formed a monophyletic clade with Southeast Asian *Microhyla* species, but some of them were very divergent from *M*. *ornata* from the type locality and likely represent yet unrecognized species (S3 Fig.). Interestingly, we also found sequences designated to *M*. *ornata* having 99% identity to the sequences of *M*. *nilphamariensis* sp. nov., and these were reported from the Western Ghats {“Bajipe, Karnoor, Talagini” as reported in [65]}. This indicates that the new species *M*. *nilphamariensis* sp. nov. might also be present in the Western Ghats of India, but is probably misidentified as *M*. *ornata* due to their close phenotypic resemblance. Therefore, further studies on the presence of the new species and its distribution in India are warranted.

The new species lacks distinctive metatarsal tubercles, which were clearly present in the *M*. *ornata* type material we examined. In addition to metatarsal tubercles, the large and pointed snout of *M*. *ornata* was highlighted as diagnostic criterion in the original description [23]. Hence, the new species is differentiated from *M*. *ornata* in both these diagnostic criteria. However, since the original author did not designate the *M*. *ornata* holotype [23], we suggest that the specimens of *M*. *ornata* from ZSI should be recognized as a series of neotypes. This is because they bear the closest morphological similarity with the original description, and also because they originate from the type locality and regions close to it in the Western Ghats of India. In addition, specimens of *M*. *nilphamariensis* sp. nov should also be identified from the museum collections as our analyses indicate the presence of *M*. *nilphamariensis* sp. in the Western Ghats, India and it is highly likely that some of the specimens now designated as “*M*. *ornata*” are actually *M*. *nilphamariensis* sp. nov.

**Fig 6:**
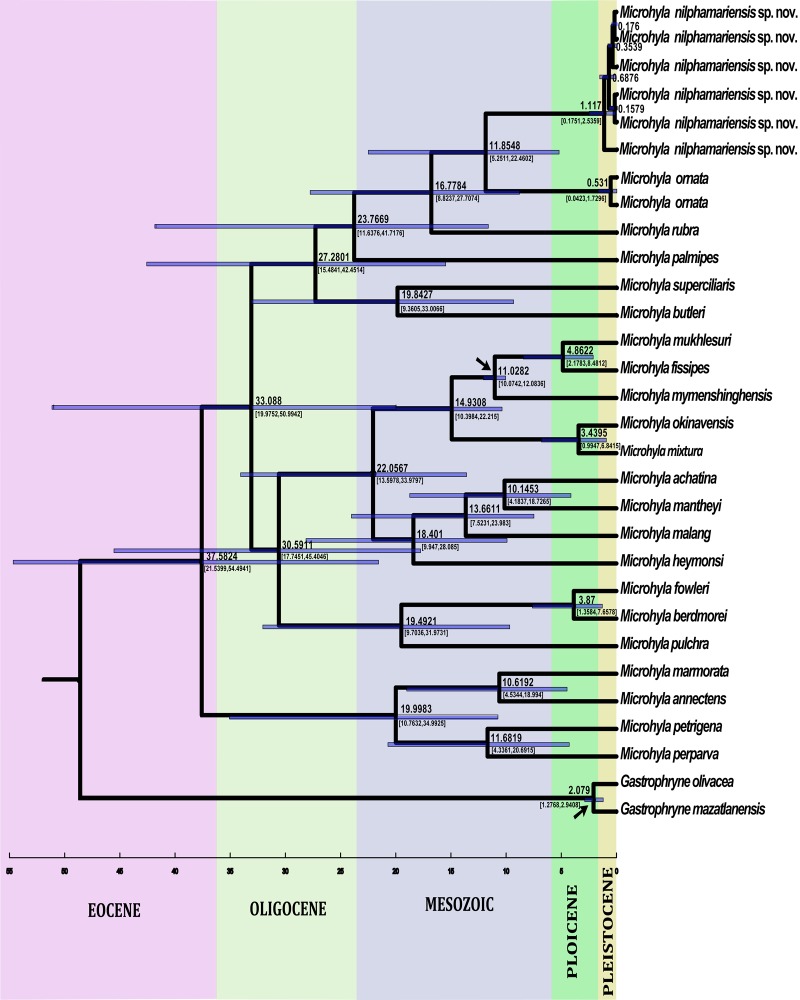
A Bayesian time-tree generated from mitochondrial 16S gene fragment for all known species in the genus *Microhyla*. Calibration points are indicated with arrows. Numbers are in million years, and the light blue colored bars indicate 95% confidence intervals for divergence time estimates.

The use of a highly divergent outgroup for phylogenetic analysis may lead to errors because as the distance from the root to the ingroup increases, the shared character states between the divergent group and ingroup taxa may not be based on history, but to chance [66]. For lack of a better alternative, we used a rather divergent outgroup from the genus *Gastrophryne* (Family Microhylidae) to calibrate one of the nodes needed for estimation of the divergence times between taxa in our phylogenetic analysis. Although the *Gastrophryne* fossil provides a reliable estimate for calibration of the divergence time, it may be problematic in being highly divergent from the ingroup taxa. Nevertheless, we also calibrated the divergence time between the ingroup nodes using the known divergence between *Microhyla fissipes* and *M*. *mymensinghensis* [10], which should add reliability to the estimates. The analyses show that the new species diverged from *M*. *ornata* about 11.85 mya (5.25 to 22.46 mya; Fig. 6), and that South Asian *Microhyla* species (*M*. *rubra*, *M*. *ornata*, and *M*. *nilphamariensis*) form a monophyletic clade distinct from all other known *Microhyla* species from this region. As India and the Bengal basin were the first to contact Southeast Asia in the early Miocene (22 mya) [67], this corresponds well with our molecular clock analyses that indicated that the South Asian Microhylids diverged from the other congeneric species about 23 mya ago.

In conclusion, we have described a new species of *Microhyla* from a highly genetically heterogeneous group of frogs that have been recognized as *M*. *ornata* for the last 173 years, due in part to the lack of detailed genetic studies. To this end, our study serves as an example of how detailed genetic and morphological comparisons among populations of species reportedly having a very broad distribution range can help in identification of yet undescribed species. This in turn can provide valuable information for assigning correct conservation status for these taxa. As to the status of *M*. *ornata*, the data we have compiled indicates that it may be endemic to the Western Ghats biodiversity hotspot: reports of *M*. *ornat*a from different regions of Asia turned out to be genetically highly divergent from the *M*. *ornata* from the actual type locality of Kerala in India. Hence, *M*. *ornata* records in literature and GeneBank may consist of several unnamed cryptic *Microhyla* species. Therefore, detailed morphological and genetic analyses would be in place to further resolve taxonomic uncertainties and identifying the cryptic diversity within this genus.

## Supporting Information

S1 Fig A schematic illustration of the definitions of morphological traits measured in this study. See Materials and methods for explanation of trait abbreviations.
(TIF)

S2 Fig Bivariate plots of some diagnostic traits *Microhyla nilphamariensis* sp. nov. and its closest relatives, *M. ornata* and *M. rubra*
(A) distance from front of eyes to the nostril (EN) *vs*. nostril–snout length (NS), (B) internarial distance (IN) *vs*. nostril–snout length (NS), (C) distance from back of mandible to back of the eye (MBE) *vs*. head length (HL), and (D) interorbital distance (IOD) *vs*. internarial distance (IN).
(TIF)

S3 Fig Maximum-likelihood phylogenetic tree based on variation in 16S gene fragment showing the position of *Microhyla nilphamariensis* sp. nov. in relation to other available *Microhyla* haplotypes from the GenBank. GenBank accession numbers and locality information is included after the scientific names. The star marked haplotype for *Microhyla ornata* is from the type locality (Kerala, India) included in the present study. The taxa indicated in red are sequences of *Microhyla* deposited in GenBank as “*Microhyla ornata*”.
(TIF)

S1 Table Additional specimens examined. (PDF)

S2 Table Gene sequences compared and deposited in GenBank. (PDF)

## References

[1] Bickford D, Lohman DJ, Sodhi NS, Ng PKL, Meier R, Winker K, et al Cryptic species as a window on diversity and conservation. Trends Ecol Evol. 2007; 22: 148–155. 17129636 10.1016/j.tree.2006.11.004

[2] Fouquet A, Gilles A, Vences M, Marty C, Blanc M, Gemmell NJ. Underestimation of species rich- ness in neotropical frogs revealed by mtDNA analyses. PLoS One. 2007; 2: e1109 17971872 10.1371/journal.pone.0001109 PMC2040503

[3] Vieites DR, Wollenberg KC, Andreone F, Köhler J, Glaw F, Vences M. Vast underestimation of Madagascar’s biodiversity evidenced by an integrative amphibian inventory. Proc Natl Acad Sci U S A. 2009; 106: 8267–8272. 10.1073/pnas.0810821106 19416818 PMC2688882

[4] Biju SD, Bocxlaer IV, Mahony S, Dinesh KP, Radhakrishnan C, Zachariah A, et al A taxonomic review of the Night Frog genus *Nyctibatrachus* Boulenger, 1882 in the Western Ghats, India (Anura: Nyctibatrachidae) with description of twelve new species. Zootaxa. 2011; 3029: 1–96.

[5] Nair A, Gopalan SV, George S, Kumar KS, Teacher AGF, Merilä J. High cryptic diversity of endemic *Indirana* frogs in the Western Ghats biodiversity hotspot. Anim Conserv. 2012; 15: 489–498.

[6] Hasan M, Islam MM, Khan MMR, Alam MS, Kurabayashi A, Igawa T, et al Cryptic anuran biodiversity in Bangladesh revealed by mitochondrial 16S rRNA gene sequences. Zoolog Sci. 2012; 29: 162–172. 10.2108/zsj.29.162 22379983

[7] Islam MM, Kurose N, Khan MMR, Nishizawa T, Kuramoto M, Alam MS, et al Genetic divergence and reproductive isolation in the genus *Fejervarya* (Amphibia: Anura) from Bangladesh inferred from morphological observation, crossing experiments, and molecular analyses. Zoolog Sci. 2008; 25: 1084–1105. 10.2108/zsj.25.1084 19267620

[8] Biju SD, Garg S, Gururaja KV, Shouche Y, Walujkar SA. DNA barcoding reveals unprecedented diversity in Dancing Frogs of India (Micrixalidae, *Micrixalus*): a taxonomic revision with description of 14 new species. Ceylon J Sci Biol Sci. 2014; 43: 37–123.

[9] Alam MS, Igawa T, Khan MMR, Islam MM, Kuramoto M, Matsui M, et al Genetic divergence and evolutionary relationships in six species of genera *Hoplobatrachus* and *Euphlyctis* (Amphibia: Anura) from Bangladesh and other Asian countries revealed by mitochondrial gene sequences. Mol Phylogenet Evol. 2008; 48: 515–527. 10.1016/j.ympev.2008.04.020 18513995

[10] Hasan M, Islam MM, Kuramoto M, Kurabayashi A, Sumida M. Description of two new species of *Microhyla* (Anura: Microhylidae) from Bangladesh. Zootaxa. 2014; 3755: 401–408. 10.11646/zootaxa.3755.5.1 24869829

[11] Matsui M, Ito H, Shimada T, Ota H, Saidapur SK, Khonsue W, et al Taxonomic relationships within the pan-oriental narrow-mouth toad *Microhyla ornata* as revealed by mtDNA analysis. Zoolog Sci. 2005; 22: 489–495. 15846058 10.2108/zsj.22.489

[12] Frost DR [Internet] Amphibian Species of the World: an Online Reference, Version 6.0.–[cited 2014 Jan 28]. Available: http://researchamnhorg/vz/herpetology/amphibia/.

[13] IUCN [Internet] IUCN Red List of Threatened Species, Version 2013.2.–[cited 2014 Feb 28]. Available: http://www.iucnredlist.org/.

[14] Boulenger GA. Descriptions of new species of reptiles and batrachians in the British Museum—Part. II. Annals and Magazine of Natural History (Series 5). 1884; 13: 396–398.

[15] Parker HW. A Monograph of the Frogs of the Family Microhylidae. London: Trustees of the British Museum; 1934.

[16] Mathew R, Sen N. Pictorial Guide to Amphibians of North East India. India: Zoological Survey of India, Kolkata; 2010.

[17] Blyth E. Report for October Meeting, 1855. Journal of the Asiatic Society of Bengal. 1856; 24: 711–723.

[18] Asmat GSM, Banu Q, Islam MA, Ahsan MF, Chakma S. Amphibian fauna from Chittagong and Chittagong Hill-tracts, Bangladesh. University Journal of Zoology, University of Rajshahi. 2003; 22: 141–143.

[19] Dutta SK, Manamendra-Arachchi K. The Amphibian Fauna of Sri Lanka. Colombo, Sri Lanka: Wildlife Heritage Trust of Sri Lanka; 1996.

[20] de Silva A. Amphibians of Sri Lanka: A Photographic Guide to Common Frogs, Toads and Caecilians. Kandy, Sri Lanka: Privately published; 2009.

[21] Dutta SK, Ray P. *Microhyla sholigari*, a new species of microhylid frog (Anura: Microhylidae) from Karnataka, India. Hamadryad. 2000; 25: 38–44.

[22] Biju SD. A synopsis of the frog fauna of the Western Ghats, India. Occasional Publication—Indian Society for Conservation Biology. 2001; 1: 1–24.

[23] Duméril AMC, Bibron G. Erpétologie Générale ou Histoire Naturelle Complète des Reptiles. Volume 8 Paris: Librarie Enclyclopedique de Roret; 1841 pp. 745–746.

[24] Jerdon TC. Catalogue of reptiles inhabiting the peninsula of India. Journal of the Asiatic Society of Bengal. 1854; 22: 522–534.

[25] Biju SD, Bossuyt F. Systematics and phylogeny of *Philautus* Gistel, 1848 (Anura, Rhacophoridae) in the Western Ghats of India, with descriptions of 12 new species. Zool J Linn Soc. 2009; 155: 374–444.

[26] Bossuyt F, Meegaskumbura M, Beenaerts N, Gower DJ, Pethiyagoda R, Roelants K, et al Local endemism within the Western Ghats-Sri Lanka biodiversity hotspot. Science. 2004; 306: 479–481. 15486298 10.1126/science.1100167

[27] Meegaskumbura M, Bossuyt F, Pethiyagoda R, Manamendra-Arachchi K, Bahir M, Milinkovitch MC, et al Sri Lanka: An amphibian hot spot. Science. 2002; 298: 379–379. 12376694 10.1126/science.298.5592.379

[28] Glaw F, Vences M. A field guide to the amphibians and reptiles of Madagascar. 3rd ed. Ko¨ ln, Germany: M. Vences & F. Glaw Verlags GbR; 2007 496 pp.

[29] Ivanova NV, deWaard JR, Hebert, PDN. An inexpensive, automation-friendly protocol for recovering high-quality DNA. Mol Ecol Resour. 2006; 6: 998–1002.

[30] Sumida M, Kondo Y, Kanamori Y, Nishioka M. Inter- and intraspecific evolutionary relationships of the rice frog *Rana limnocharis* and the allied species *R*. *cancrivora* inferred from crossing experiments and mitochondrial DNA sequences of the 12S and 16S rRNA genes. Mol Phylogenet Evol. 2002; 25: 293–305. 12414311 10.1016/s1055-7903(02)00243-9

[31] Drummond AJ, Ashton B, Buxton S, Cheung M, Cooper A, Duran C, et al. [Internet] Geneious v5.0.4.–[cited 2013 Jan 24]. Available: http://www.geneious.com.

[32] Thompson JD, Higgins DG, Gibson TJ. CLUSTAL W: Improving the sensitivity of progressive multiple sequence alignment through sequence weighting, position-specific gap penalties and weight matrix choice. Nucleic Acids Res. 1994; 22: 4673–4680. 7984417 10.1093/nar/22.22.4673 PMC308517

[33] Hall TA. BioEdit: a user-friendly biological sequence alignment editor and analysis program for Windows 95/98/NT. Nucleic Acids Symp Ser. 1999; 41: 95–98.

[34] Tamura K, Peterson D, Peterson N, Stecher G, Nei M, Kumar S. MEGA5: molecular evolutionary genetics analysis using maximum likelihood, evolutionary distance, and maximum parsimony methods. Mol Biol Evol. 2011; 28: 2731–2739. 10.1093/molbev/msr121 21546353 PMC3203626

[35] Felsenstein J. Confidence limits on phylogenies, an approach using bootstrap. Evolution. 1985; 39: 783–791. 28561359 10.1111/j.1558-5646.1985.tb00420.x

[36] Ronquist F, Huelsenbeck JP. MrBayes 3: Bayesian phylogenetic inference under mixed models. Bioinformatics. 2003; 19: 1572–1574. 12912839 10.1093/bioinformatics/btg180

[37] Sanchiz B. Encyclopedia of paleoherpetology. Part 4 Salientia. München: Verlag Dr. Friedrich Pfeil; 1998 275pp.

[38] Holman JA. Fossil frogs and toads of North America. Bloomington: Indiana University Press; 2003 246pp.

[39] Bain RH, Nguyen TQ. Three new species of narrow-mouthed frogs (genus Microhyla) from Indochina, with comments on *Microhyla annamensi*s and *Microhyla palmipes* . Copeia. 2004; 2004: 507–524.

[40] Boulenger GA. Descriptions of new batrachians and reptiles from the Larut Hills, Perak. Annals and Magazine of Natural History (Series 7). 1900; 6: 186–193.

[41] Boulenger GA. Descriptions of new Malay frogs. Annals and Magazine of Natural History (Series 6). 1897; 19: 106–108.

[42] Das I, Yaakob NS, Sukumaran J. A new species of *Microhyla* (Anura: Microhylidae) from the Malay Peninsula. Hamadryad. 2007; 31: 304–314.

[43] Guibé J. Catalogue des Types d'Amphibiens du Muséum National d'Histoire Naturelle. Paris: Imprimerie Nationale; 1950.

[44] Hu S.-q. Zhao E.-m., Liu C.-c. A herpetological survey of the Tsinling and Ta-Pa Shan region. Acta Zoologica Sinica/ Dong wu xue bao. 1966; 18: 57–89. [In Chinese with English abstract].

[45] Inger RF. Four new species of frogs from Borneo. Malayan Nature Journal. 1989; 42: 229–243.

[46] Inger RF, Frogner KJ. New species of narrow-mouth frogs (genus *Microhyla*) from Borneo. Sarawak Museum Journal. 1979; 27: 311–322.

[47] Matsui M. Taxonomic revision of one of the Old World’s smallest frogs, with description of a new Bornean *Microhyla* (Amphibia, Microhylidae). Zootaxa. 2011; 2814: 33–49.

[48] Matsui M, Hamidy A, Eto K. Description of a new species of *Microhyla* from Bali, Indonesia (Amphibia, Anura). Zootaxa. 2013; 3670: 579–590. 26438961 10.11646/zootaxa.3670.4.9

[49] Parker HW. The brevicipitid frogs of the genus *Microhyla* . Annals and Magazine of Natural History (Series 10). 1928; 2: 473–499.

[50] Pillai RS. On two frogs of the family Microhylidae from Andamans including a new species. Proceedings of the Indian Academy of Sciences, Section-B. 1977; 86: 135–138.

[51] Smith MA. Notes on reptiles and batrachians from Siam and Indo-China (no. 2). Journal of the Natural History Society of Siam. 1923; 6: 47–53.

[52] Taylor EH. Zoological results of the third De Schauensee Siamese Expedition, Part III. Amphibians and reptiles. Proceedings of the Academy of Natural Sciences of Philadelphia. 1934; 86: 281–310.

[53] Tschudi J Jv. Classification der Batrachier mit Berücksichtigung der fossilen Thiere dieser Abtheilung der Reptilien. Neuchâtel: Petitpierre; 1838.

[54] Vogt T. Beitrag zur Amphibien-fauna der Insel Formosa. Sitzungsberichte der Gesellschaft Naturforschender Freunde zu Berlin. 1911; 1911: 179–184.

[55] Poyarkov NA Jr, Vassilieva AB, Orlov NL, Galoyan EA, Tran DTA, Le DTT, et al Taxonomy and distribution of narrow-mouth frogs of the genus Microhyla Tschudi, 1838 (Anura: Microhylidae) from Vietnam with descriptions of five new species. Russ J Herpetol. 2014; 21: 89–148

[56] Schenkel E. Achter Nachtrag zum Katalog der herpetologischen Sammlung des Basler Museums. Verhandlungen der Naturforschenden Gesellschaft in Basel. 1901; 13: 142–199.

[57] Andersson LG. A small collection of frogs from Annam collected in the years 1938–1939 by Bertil Björkegren. Arkiv för Zoologi. 1942; 34: 1–11. 6349588

[58] Hallowell E. Report upon the Reptilia of the North Pacific Exploring Expedition, under command of Capt. John Rogers, U.S. N. Proceedings of the Academy of Natural Sciences of Philadelphia. 1861; 12: 480–510.

[59] Tarkhnishvili DN. Amphibian communities of the southern Vietnam: Preliminary data. Journal of the Bengal Natural History Society, New Series. 1994; 13: 3–62.

[60] Fernando P, Siriwardhane M. *Microhyla karunaratnei* (Anura: Microhylidae), a new species of frog endemic to Sri Lanka. Journal of South Asian Natural History. 1996; 2: 135–142.

[61] Parker HW, Osman-Hill WC. Frogs of the genus *Microhyla* from Ceylon. Annals and Magazine of Natural History (Series 12). 1949; 1: 759–764.

[62] Bossuyt F, Milinkovitch MC. Convergent adaptive radiations in Madagascan and Asian ranid frogs reveal covariation between larval and adult traits. Proc Natl Acad Sci U S A. 2000; 97: 6585–6590. 10841558 10.1073/pnas.97.12.6585 PMC18667

[63] Emerson SB. Heterochrony and frogs: the relationship of a life history trait to morphological form. Am Nat. 1986; 127: 167–183.

[64] Kuramoto M, Joshy SH. Morphological and acoustic comparisons of *Microhyla ornata*, *M*. *fissipes*, and *M*. *okinavensis* (Anura: Microhylidae). Curr Herpetol. 2006; 25: 15–27.

[65] Hasan M, Islam MM, Khan MMR, Igawa T, Alam MS, Djong TH, et al Genetic divergences of South and Southeast Asian frogs: A case study of several taxa based on 16S ribosomal RNA gene data with notes on the generic name *Fejervarya* . Turk Zool Derg. 2014; 38: 389–411.

[66] Wheel WC. Nucleic acid sequence phylogeny and random outgroups. Cladistics. 1990; 6: 363–367. 10.1111/j.1096-0031.1990.tb00550.x 34933486

[67] Alam M, Alam MM, Curray JR, Chowdhury M, Gani MR. An overview of the sedimentary geology of the Bengal Basin in relation to the regional tectonic framework and basin-fill history. Sediment Geol. 2003; 155: 179–208.

