# Supplementary figures and images for: A New Species of Microhyla (Anura: Microhylidae) from Nilphamari, Bangladesh
Source: PLoS One. 2015 Mar 25;10(3):e0119825. doi: 10.1371/journal.pone.0119825 (PMC4373918; doi:10.1371/journal.pone.0119825)

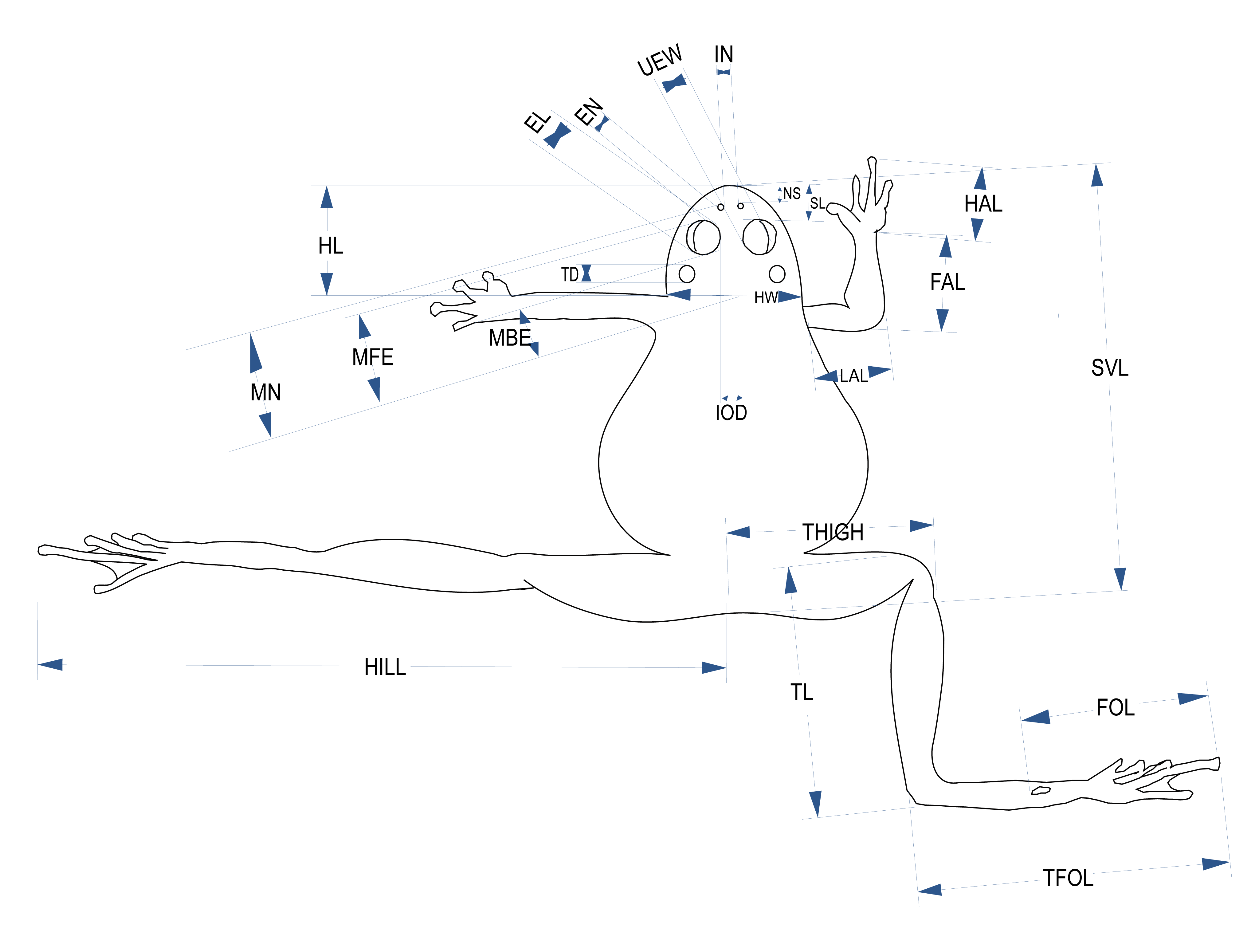

Supplement: S1 Fig — See Materials and methods for explanation of trait abbreviations. (TIF) [file pone.0119825.s001.tif]

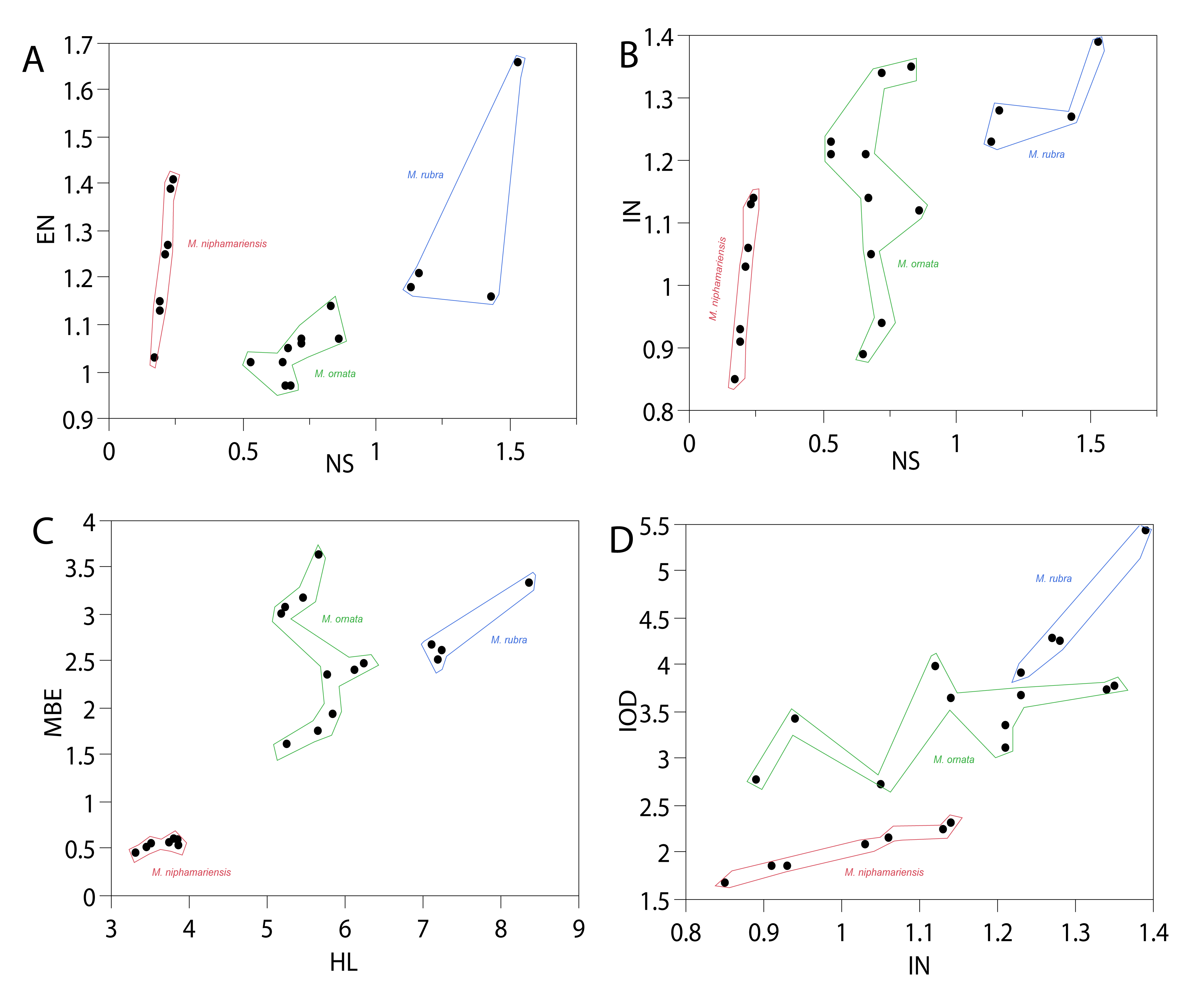

Supplement: S2 Fig — (A) distance from front of eyes to the nostril (EN) vs. nostril–snout length (NS), (B) internarial distance (IN) vs. nostril–snout length (NS), (C) distance from back of mandible to back of the eye (MBE) vs. head length (HL), and (D) interorbital distance (IOD) vs. internarial distance (IN). (TIF) [file pone.0119825.s002.tif]

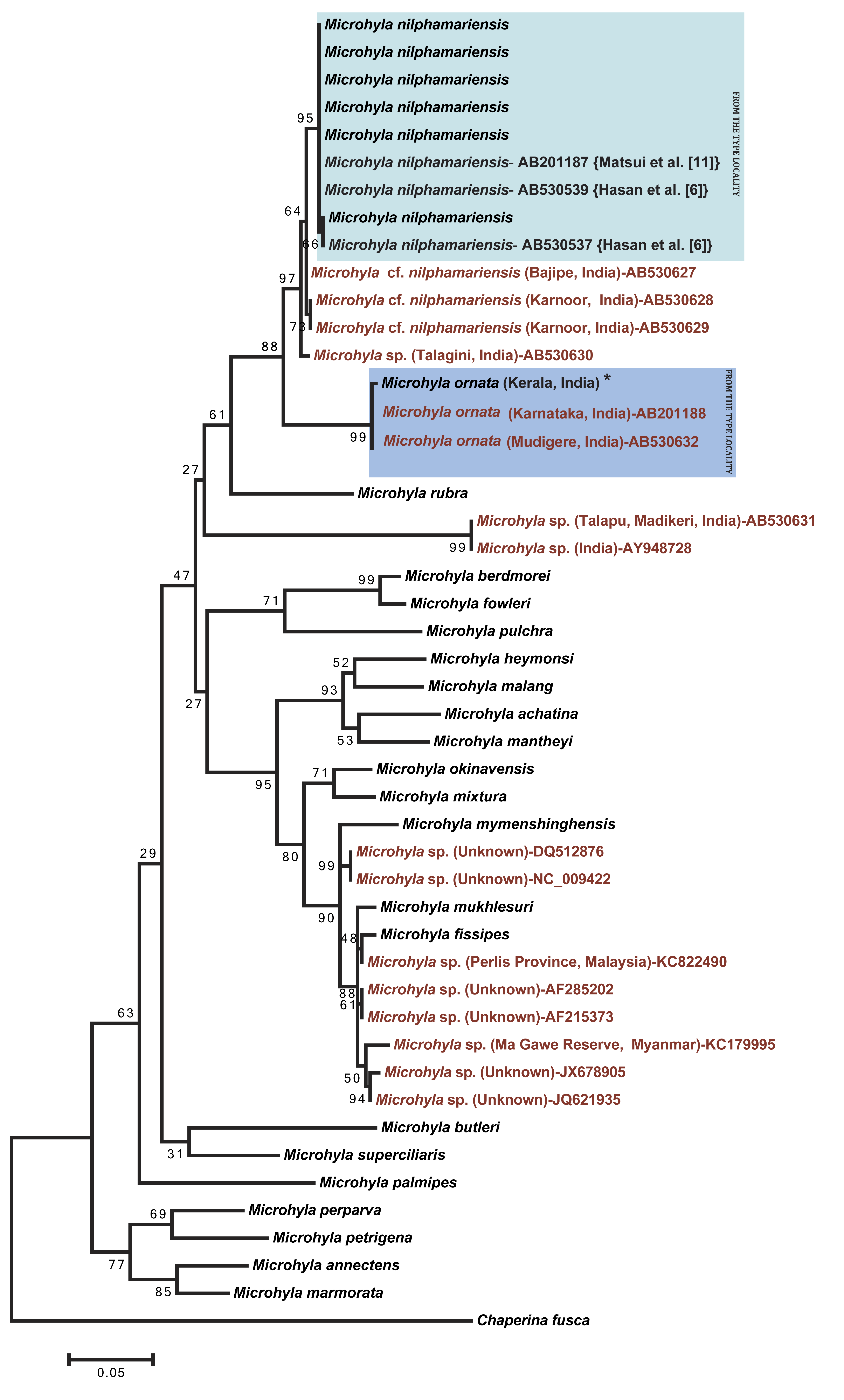

Supplement: S3 Fig — GenBank accession numbers and locality information is included after the scientific names. The star marked haplotype for Microhyla ornata is from the type locality (Kerala, India) included in the present study. The taxa indicated in red are sequences of Microhyla deposited in GenBank as “Microhyla ornata”. (TIF) [file pone.0119825.s003.tif]
