# Supplementary material for: A New Species of Microhyla (Anura: Microhylidae) from Nilphamari, Bangladesh
Source: PLoS One. 2015 Mar 25;10(3):e0119825. doi: 10.1371/journal.pone.0119825 (PMC4373918; doi:10.1371/journal.pone.0119825)
Supplement: S1 Table — (PDF) [file pone.0119825.s004.pdf]

S1 Table. Additional specimens examined.

***Microhyla ornata* (10 specimens):** Zoological Survey of India. ZSI A9080/1– A9080/10. **Collected from:** Lukunda estate, Vigarpet District, Srimangala Nadu, South Coorg, Karnataka, India.

***Microhyla rubra* (5 specimens):** Zoological Survey of India. ZSI A9110, **Collected from:** Lukunda estate, Vigarpet District, Srimangala Nadu, South Coorg, Karnataka, India. ZSI A10810, ZSI A10810/1, ZSI A10810/2, **Collected from**: In and Around Murkal Forest Rest House, Karnataka, India.

***Microhyla sholigari* (5 specimens**): Zoological Survey of India: ZSI A9061– A9065. **Collected from:** Biligirirangan Hills, Chamrajanagar District, Yelandur Taluk, Karnataka, India.
