# Supplementary material for: A New Species of Microhyla (Anura: Microhylidae) from Nilphamari, Bangladesh
Source: PLoS One. 2015 Mar 25;10(3):e0119825. doi: 10.1371/journal.pone.0119825 (PMC4373918; doi:10.1371/journal.pone.0119825)
Supplement: S2 Table — (PDF) [file pone.0119825.s005.pdf]

S2 Table. Gene sequences compared and deposited in GenBank.

Species	Voucher	Collection locality	GenBank accession numbers (*16S rRNA*)	Reference
Compared gene sequences				
Microhyla ornata	ZSI-A9119	Dhawad, Karnataka, India	AB201188	[1]
Microhyla butleri	KUHE33557	Bangkok, Thailand	AB201189	[1]
Microhyla okinavensis	IABHU5263	------------------	AB303950	[2]
Microhyla fissipes	KUHE35165	Kanchanaburi, Thailand	AB201186	[1]
Microhyla heymonsi	--------------	------------------	AY458596	[3]
Microhyla perparva	---------------	Kubah National Park, Malaysia	GU154884	[4]
Microhyla rubra	---------------	Dhawad, Kerala, India	AB201192	[1]
Microhyla berdmorei	KUHE 52034	Gombak, Malaysia	AB598338	[5]
Microhyla mixtura	CIB 20070248	China, Sichuan	AB634669	[5]
Microhyla malang	KUHE 42597	Kanowit, Sibu Division, Malaysia	AB598322	[6]
Microhyla achatina	MZB Amp 16401	Ungaran, Java, Indonesia	AB598335	[6]
Microhyla mantheyi	KUHE 52556	Temerloh, Pahang, Malaysia	AB598334	[6]
Microhyla pulchra	KUHE35119	Kanchanaburi, Thailand	AB201191	[1]
Microhyla superciliaris	KUHE 52558	Pahang, Temerloh, Malaysia	AB634682	[5]
Microhyla fowleri	KUHE 21992	Phrae, Mae Yom, Thailand	AB634667	[5]
Microhyla annectens	KUHE 52438	Pahang, Cameron, Malaysia	AB634659	[5]
Microhyla palmipes	MZB Amp 16323	Sumatra,Bengkulu, Indonesia	AB634671	[5]
Microhyla marmorata	KUHE 32455	Houapan, Xamneua, Laos	AB611955	[7]
Microhyla petrigena	KUHE 53743	Sarawak, Bukit Kana, Malaysia	AB634675	[5]
Microhyla mukhlesuri	IABHU3880	Raozan, Chittagong, Bangladesh	AB543609	[8]
Microhyla mymensinghensis	IABHU3899	Golapganj, Sylhet, Bangladesh	AB543607	[8]
Chaperina fusca (outgroup)	FMNH 231111	Sabah, Danum Valley, Research Center, Malaysia	DQ283145	[9]
Gastrophryne olivacea	UTA A-60976	Kleberg Co., Texas, USA	JQ268514	[10]
Gastrophryne mazatlanensis	MVZ 228275	Santa Cruz Co., Arizona, USA	JQ268527	[10]
Deposited gene sequences				
Microhyla nilphamariensis	MZH-2360-66	Saidpur, Bangladesh	KP072787, KP072788, KP072789, KP072790, KP072791, KP072792, KP072793	[Present study]
Microhyla ornata	RGCB15059	Pulpally, Wayanad District, Kerala, India	KP072794	[Present study]

Museum abbreviations: CIB (Chengdu Institute of Biology, Chinese Academy of Sciences), FMNH (Field Museum, Chicago), IABHU (Institute for Amphibian Biology, Graduate School of Science, Hiroshima University), KUHE (Kyoto University, Graduate School of Human and Environmental Studies), MZB (Museum Zoologicum Bogoriense), MZD (Finnish Museum of Natural History), and RGCB (Rajiv Gandhi Centre for Biotechnology), UTA (University of Texas at Arlington) and MVZ, (Museum of Vertebrate Zoology, USNM FS, Smithsonian Institute Natural History Museum Field Series).

References:

1. Matsui M, Ito H, Shimada T, Ota H, Saidapur SK, Khonsue W, et al. Taxonomic relationships within the pan-oriental narrow-mouth toad *Microhyla ornata* as revealed by mtDNA analysis. Zoological Science. 2005; 22: 489–495.
2. Igawa T, Kurabayashi A, Usuki C, Fujii T, Sumida M. Complete mitochondrial genomes of three neobatrachian anurans: a case study of divergence time estimation using different data and calibration settings. Gene. 2008; 407: 116–129.
3. Zhang P, Zhou H, Chen YQ, Liu YF, Qu LH. Mitogenomic Perspectives on the Origin and Phylogeny of Living Amphibians. Systematic Biology. 2005; 54: 391–400.
4. Das I, Haas A. New species of *Microhyla* from Sarawak: Old World's smallest frogs. Zootaxa. 2010; 2571: 37–52
5. Matsui M, Hamidy A, Belabut DM, Ahmad N, Panha S, Sudin A, et al. Systematic relationships of Oriental tiny frogs of the family Microhylidae (Amphibia, Anura) as revealed by mtDNA genealogy. Molecular Phylogenetics and Evolution. 2011; 61: 167–176.
6. Matsui M. Taxonomic revision of one of the Old World's smallest frogs, with description of a new Bornean *Microhyla* (Amphibia, Microhylidae). Zootaxa. 2011; 2814: 33–49.
7. Kurabayashi A, Matsui M, Belabut DM, Yong HS, Ahmad N. From Antarctica or Asia? New colonization scenario for Australian-New Guinean narrow mouth toads suggested from the findings on a mysterious genus *Gastrophrynoides*. BMC Evolutionary Biology. 2011; 11:175. doi:10.1186/1471-2148-11-175.
8. Hasan M, Islam MM, Khan MMR, Alam MS, Kurabayashi A, Igawa T, et al. Cryptic anuran biodiversity in Bangladesh revealed by mitochondrial 16S rRNA gene sequences. Zoological Science. 2012; 29: 162–172.
9. Frost DR, Grant T, Faivovich J, Bain RH, Haas A, Haddad CFB, et al. The amphibian tree of life. Bulletin of the American Museum of Natural History. 2006; 297: 1–370.
10. Streicher JW, Cox CL, Campbell JA, Smith EN and de Sa RO. Rapid range expansion in the Great Plains narrow-mouthed toad (*Gastrophryne olivacea*) and a revised taxonomy for North American microhylids. Molecular Phylogenetics and Evolution, 2012; 64: 645–653.
